# Potential Therapeutic Target and Vaccines for SARS-CoV-2

**DOI:** 10.3390/pathogens12070926

**Published:** 2023-07-10

**Authors:** Mohamed A. Hussain, Mohamed M. Hassan, Bashir Abdrhman Bashir, Tarig A. Gamar, Elmuaiz Gasmalbari, Ahmed Osman Mohamed, Wadah Osman, Asmaa E. Sherif, Abdelaziz Elgaml, Aisha A. Alhaddad, Kholoud F. Ghazawi, Samar F. Miski, Bayan E. Ainousah, Yusra Saleh Andijani, Sabrin R. M. Ibrahim, Gamal A. Mohamed, Ahmed Ashour

**Affiliations:** 1 Department of Pharmaceutical Microbiology, Faculty of Pharmacy, International University of Africa, Khartoum 11111, Sudan; ahmedkunna93@hotmail.com; 2 Department of Hematology, Faculty of Medical Laboratory Science, National University, Khartoum 11111, Sudan; mhassan0210@gmail.com; 3 Department of Hematology, Faculty of Medical Laboratory Sciences, Port Sudan Ahlia College, Port Sudan 33312, Sudan; bashirbashir17@hotmail.com; 4 Department of Medical Parasitology, Faculty of Medical Laboratory Sciences, University of Sciences and Technology, Khartoum North 13311, Sudan; tariqquamer35@yahoo.com; 5 Faculty of Medicine, Omdurman Islamic University, Al Khartoum 14415, Sudan; elmuaizcovid19@gmail.com; 6 Department of Pharmacognosy, Faculty of Pharmacy, Prince Sattam Bin Abdulaziz University, Al-Kharj 11942, Saudi Arabia; w.osman@psau.edu.sa (W.O.); asmaasherif80@mans.edu.eg (A.E.S.); ahmed.mohamed@psau.edu.sa (A.A.); 7 Department of Pharmacognosy, Faculty of Pharmacy, University of Khartoum, Al-Qasr Ave, Khartoum 11111, Sudan; 8 Department of Pharmacognosy, Faculty of Pharmacy, Mansoura University, Mansoura 35511, Egypt; 9 Microbiology and Immunology Department, Faculty of Pharmacy, Mansoura University, Mansoura 35511, Egypt; elgamel3a@mans.edu.eg; 10 Microbiology and Immunology Department, Faculty of Pharmacy, Horus University, New Damietta 34517, Egypt; 11 Department of Pharmacology and Toxicology, College of Pharmacy, Taibah University, Al-Madinah Al-Munawwarah 30078, Saudi Arabia; aahaddad@taibahu.edu.sa (A.A.A.); smiski@taibahu.edu.sa (S.F.M.); yandijani@taibahu.edu.sa (Y.S.A.); 12 Clinical Pharmacy Department, College of Pharmacy, Umm Al-Qura University, Makkah 24382, Saudi Arabia; kfghazawi@uqu.edu.sa; 13 Department of Pharmaceutical Chemistry, Faculty of Pharmacy, Umm Al-Qura University, Makkah 21955, Saudi Arabia; baaunosah@uqu.edu.sa; 14 Preparatory Year Program, Department of Chemistry, Batterjee Medical College, Jeddah 21442, Saudi Arabia; sabrin.ibrahim@bmc.edu.sa; 15 Department of Pharmacognosy, Faculty of Pharmacy, Assiut University, Assiut 71526, Egypt; 16 Department of Natural Products and Alternative Medicine, Faculty of Pharmacy, King Abdulaziz University, Jeddah 21589, Saudi Arabia; gahussein@kau.edu.sa

**Keywords:** comparative genomics, coronavirus, COVID-19, genes and proteins analysis, therapeutic target and vaccine, SDGs, health and well-being

## Abstract

The coronavirus has become the most interesting virus for scientists because of the recently emerging deadly SARS-CoV-2. This study aimed to understand the behavior of SARS-CoV-2 through the comparative genomic analysis with the closest one among the seven species of coronavirus that infect humans. The genomes of coronavirus species that infect humans were retrieved from NCBI, and then subjected to comparative genomic analysis using different bioinformatics tools. The study revealed that SARS-CoV-2 is the most similar to SARS-CoV among the coronavirus species. The core genes were shared by the two genomes, but there were some genes, found in one of them but not in both, such as ORF8, which is found in SARS-CoV-2. The ORF8 protein of SARS-CoV-2 could be considered as a good therapeutic target for stopping viral transmission, as it was predicted to be a transmembrane protein, which is responsible for interspecies transmission. This is supported by the molecular interaction of ORF8 with both the ORF7 protein, which contains a transmembrane domain that is essential to retaining the protein in the Golgi compartment, and the S protein, which facilitates the entry of the coronavirus into host cells. ORF1ab, ORF1a, ORF8, and S proteins of SARS-CoV-2 could be immunogenic and capable of evoking an immune response, which means that these four proteins could be considered a potential vaccine source. Overall, SARS-CoV-2 is most related to SARS-CoV. ORF8 could be considered a potential therapeutic target for stopping viral transmission, and ORF1ab, ORF1a, ORF8, and the S proteins of SARS-CoV-2 could be utilized as a potential vaccine source.

## 1. Introduction

The recently established SDGs (Sustainable Development Goals) in 2015 aim to address the systemic barriers to social, economic, and environmentally sustainable development with a universal application under the premise of an interconnected, growing world [1]. Since the adoption of the SDGs, numerous governments, UN agencies, and regional and international organizations have taken great steps to implement this ambitious global framework [1,2]. However, the emergency of the coronavirus disease 2019 (COVID-19) pandemic has posed a significant challenge to achieving the SDGs, which are aimed to be achieved by 2030 [2].

Coronaviruses (CoVs) are enveloped in single-stranded positive-sense RNA viruses [3]. On the basis of phylogenetic analyses and antigenic criteria, coronaviruses have been divided into four classes: alphacoronavirus, betacoronavirus, gammacoronavirus, and deltacoronavirus [4,5].

For the first time in the year 1960, both in adults and children, human coronavirus was identified as a result of respiratory infection [6]. High scientific interest in CoV studies only arose when the first severe acute respiratory syndrome (SARS-CoV) appeared in 2002 [7,8]. Due to the global spread of SARS-CoV, approximately 8000 confirmed human cases and 774 deaths (approximately a 9.5 percent mortality rate) occurred [9,10]. In 2012 Middle East respiratory syndrome CoV (MERS-CoV) outbreak in Saudi Arabia heightened this interest, owing to the higher mortality rate (approximately 35%) compared to SARS-CoV [11].

SARS-CoV-2, a novel betacoronavirus detected in the Chinese province of Wuhan, has recently been linked to severe respiratory infections in humans. The global spread of SARS-CoV-2, with a high risk of human-to-human transmission, prompted the World Health Organization to declare a public health emergency of international concern on 30 January 2020. After that, the virus spread rapidly beyond China, and the WHO declared the coronavirus disease (COVID-19) a pandemic on 11 March 2020 [12]. More than 655 million confirmed COVID-19 cases, with over 6.5 million deaths worldwide, had been reported by 15 December 2022 [13].

Coronavirus genomes are the largest among RNA viruses, ranging from 26 to 32 kilobases in size. These genomes have four major structural proteins: the spike (S), membrane (M), envelope (E), and nucleocapsid (N). The S protein mediates the virus’s attachment to host cell surface receptors, resulting in fusion and subsequent viral entry. The M protein defines the shape of the viral envelope and is the most abundant protein [14]. The E protein is the smallest of the major structural proteins and participates in viral assembly and budding. The N protein is the only one that binds to the RNA genome and is involved in viral assembly and budding [15,16]. Coronaviruses have a number of nonstructural and accessory proteins, including Orf1ab, Orf3a, Orf6, Orf7a, Orf10, and Orf8 [17,18]. If their structures are characterized and their mechanisms of action and roles in viral replication are recognized, this will result in an increase in the number of suitable therapeutic targets [15]. Among nonstructural proteins, researchers have paid more attention to the Orf8 protein because it enhances viral replication and affects DNA synthesis and degradation of E proteins [17].

Coronaviridae members implicated in human infection show several similarities regarding genome structure [19]. Therefore, the aim of this study was to understand the behavior of SARS-CoV-2 through comparative genomic analysis with the closest one among the seven species of coronavirus that infect humans. The achievement of our aims may provide clues for ongoing and future research efforts regarding the understanding and containment of SARS-CoV-2.

## 2. Material and Methods

### 2.1. Whole Genomes Analysis

Eight genomes of coronaviruses that implicated human infection were retrieved from the Nucleotide database (https://www.ncbi.nlm.nih.gov/nuccore, accessed on 15 December 2022), one of the National Center for Biotechnology Information’s subdivided databases (NCBI: https://www.ncbi.nlm.nih.gov/, accessed on 15 December 2022). The names of the targeted genomes and their accession numbers are as follows; SARS-CoV (NC_004718.3: https://www.ncbi.nlm.nih.gov/nuccore/NC_004718.3/, accessed on 15 December 2022), MERS (NC_019843.3: https://www.ncbi.nlm.nih.gov/nuccore/NC_019843.3, accessed on 15 December 2022), OC43 (NC_006213.1: https://www.ncbi.nlm.nih.gov/nuccore/NC_006213.1/, accessed on 15 December 2022), 4408 (FJ415324.1: https://www.ncbi.nlm.nih.gov/nuccore/FJ415324.1/, accessed on 15 December 2022), HKU1 (NC_006577.2: https://www.ncbi.nlm.nih.gov/nuccore/NC_006577.2/, accessed on 20 December 2022), SARS-CoV-2 (NC_045512.2: https://www.ncbi.nlm.nih.gov/nuccore/NC_045512.2/, accessed on 20 December 2022), 229E (NC_002645.1: https://www.ncbi.nlm.nih.gov/nuccore/NC_002645.1/, accessed on 20 December 2022), and NL63 (NC_005831.2: https://www.ncbi.nlm.nih.gov/nuccore/NC_005831.2/, accessed on 20 December 2022). Genomic pairwise and multiple sequence alignments (MSA) were computed by using CLC Genomics Workbench 20 (https://digitalinsights.qiagen.com/, accessed on 25 December 2022). MSA was performed based on multiple sequence comparison by log-expectation (MUSCLE) algorithm [20]. Both the previous and upcoming steps were used to compare the sequences, discover similarities, differences, and evolutionary distance. The evolutionary trees were constructed using the neighbor-joining, UPGMA, minimum evolution, maximum likelihood, and maximum parsimony methods in MEGA7 (molecular evolutionary genetics analysis) software version 7.0 for larger datasets (https://www.megasoftware.net/, accessed on 25 December 2022) [21]. Bootstrap statistic method was used for each method of tree construction to show the confidence levels of branching or building the evolutionary trees. Bootstrapping values reflect how many times out of 100 the same branch appeared, while the phylogenetic analysis was replicated [22].

### 2.2. Comparative Genomic Analysis of SARS-CoV-2 with the Most Relevant One

These steps were used to compare SARS-CoV-2 with the closest one (based on phylogenetic analysis). In the beginning, GeneCo software was used to analyze multiple genome structures by using Genebank format as an input file (https://bigdata.dongguk.edu/geneCo/#/index/main, accessed on 15 January 2023). Then, nucleotide sequence statistics (general sequence information, counts of atoms, nucleotide frequencies, and comparison elements) were generated using CLC Genomics Workbench 20. Pairwise alignment between the two genomes was performed to explore conservation of synteny, in the context of the entire sequences and their annotation by using ACT: the Artemis Comparison Tool (http://sanger-pathogens.github.io/Artemis/ACT/, accessed on 15 January 2023) [23], and BLAST: Basic Local Alignment Searching Tool (https://blast.ncbi.nlm.nih.gov/Blast.cgi, accessed on 15 January 2023).

### 2.3. Low Similarity Region Analysis

There were three regions of low similarity, regions 1 and 2 contain similar genes in both genomes, whereas region 3 contains genes that are specific for each genome. Analysis of these regions was divided into two parts. The first one was for regions 1 and 2, and the second was for region 3.

Concerning similar genes/proteins within two compared genomes, identity, difference, number of gaps, and evolutionary distance were calculated using CLC Genomics Workbench 20.0 and MEGA version 7. PROFphd software (PredictProtein server) was used for conversion of primary to secondary protein structures (https://predictprotein.org/, accessed on 15 January 2023) [24]. Homology modeling for tertiary structure of spike proteins was performed using SWISS-Model server [25]. Building a homology model embraces four main steps: (i) identification of structural template(s), (ii) alignment of target sequence and template structure(s), (iii) model-building, and (iv) model quality evaluation. Each model is evaluated with three methods as follows: quaternary structure quality estimate QSQE (a score is a number between 0 and 1, the larger number is better), global model quality estimation GMQE (the score is expressed as a number between 0 and 1, larger numbers indicate higher reliability), and qualitative model energy analysis (QMEAN), which is a composite estimator based on different geometrical properties and provides both global (for the entire structure) and local (per residue) absolute quality estimates on the basis of one single model. For models with greater than 100 residues, the QMEAN score must be greater than −5. SWISS-Model server is available at: https://swissmodel.expasy.org/, accessed on 15 January 2023. After that, TM-align algorithm (https://zhanglab.ccmb.med.umich.edu/TM-align/, accessed on 15 January 2023) was used to compare the spike protein structures of SARS-CoV and SARS-CoV-2 of unknown equivalence [26]. An optimal superposition of the two structures was built on the detected alignment was returned, as well as the TM-score value, which scales the structural similarity. TM-score has a value between 0 and 1, where 1 indicates a perfect match between two structures. Scores below 0.2 correspond to randomly chosen unrelated proteins, while those higher than 0.5 assume generally the same fold, based on SCOP and CATH, respectively, which are the two most prominent protein structure classification schemes [27]. Furthermore, the antigenicity of all proteins in regions 1 and 2 was predicted for two reasons, firstly, as a comparative factor, and secondly, to predict the protective antigens. Antigenicity was predicted using a couple of tools: a commercial CLC Genomics Workbench 20 that displays the results as a plot and the publicly available VaxiJen version 2.0 (http://www.ddg-pharmfac.net/vaxijen/VaxiJen/VaxiJen.html, accessed on 20 January 2023), which provides the findings as an overall prediction score. VaxiJen has a threshold for each model (virus, bacteria, parasite, fungal, or tumor), score below the threshold will be predicted as nonantigen, and if higher, it will be predicted as antigen.

Regarding region 3, which contains genes that are specific to each genome, there is no scope for comparison. As these proteins are hypothetical, they were first subjected to comparison with the proteins in the Universal Protein Resource (UniProt: https://www.uniprot.org/, accessed on 20 January 2023) by using the BLASTp algorithm.

Due to the lack of data within the main databases (NCBI and UniProt), other tools were used to predict a variety of information about their properties, functions, structures, etc. PredictProtein server was used to predict proteins secondary structures. Proteins structure features and annotations were predicted using PSIPRED server (http://bioinf.cs.ucl.ac.uk/psipred/, accessed on 20 January 2023) [28]. Furthermore, MEMSAT-SVM tool (available within the PSIPRED server) was used to predict transmembrane protein topology [29]. This method is capable of differentiating signal peptides from transmembrane helices. Then, many algorithms and databases were used for the prediction of more information about proteins’ functions: Pfam (http://pfam.xfam.org/, accessed on 20 January 2023), InterPro (https://www.ebi.ac.uk/interpro/, accessed on 20 January 2023), Conserved Domains database (https://www.ncbi.nlm.nih.gov/cdd, accessed on 20 January 2023), PANDA (http://dna.cs.miami.edu/PANDA/, accessed on 20 January 2023), and Prosite database (https://prosite.expasy.org/, accessed on 20 January 2023). In addition, Virus-mPLoc (http://www.csbio.sjtu.edu.cn/bioinf/, accessed on 20 January 2023), and CELLO2GO (http://cello.life.nctu.edu.tw/cello2go/, accessed on 20 January 2023), were used for prediction of subcellular location of these proteins. VaxiJen v2.0 (virus model selected) was used for prediction of antigenicity. For evidence of molecular interactions of target proteins, IntAct Database (https://www.ebi.ac.uk/intact/, accessed on 20 January 2023) was used.

For the prediction of protein structures in the third region, the Swiss-Model server was used because it uses the homology modeling method, which is the most accurate when the target and template have similar sequences. Due to the lack of structural data for these proteins, additional servers with different based methods were used: DMPfold (http://bioinf.cs.ucl.ac.uk/psipred/, accessed on 20 January 2023), I-TASSER (https://zhanglab.ccmb.med.umich.edu/I-TASSER/, accessed on 20 January 2023), and Robetta (https://robetta.bakerlab.org/, accessed on 20 January 2023). Finally, PROSESS (protein structure evaluation suite and server) was used to evaluate and validate protein structures. PROSESS integrates a variety of previously developed, well-known, and thoroughly tested methods to evaluate both global and residue-specific quality: (i) covalent and geometric quality; (ii) nonbonded/packing quality; (iii) torsion angle quality; (iv) chemical shift quality, and (v) NOE quality. Server available at: http://www.prosess.ca/index.php, accessed on 20 January 2023).

## 3. Results and Discussion

### 3.1. Whole Genome Analysis

**Figure 1:**
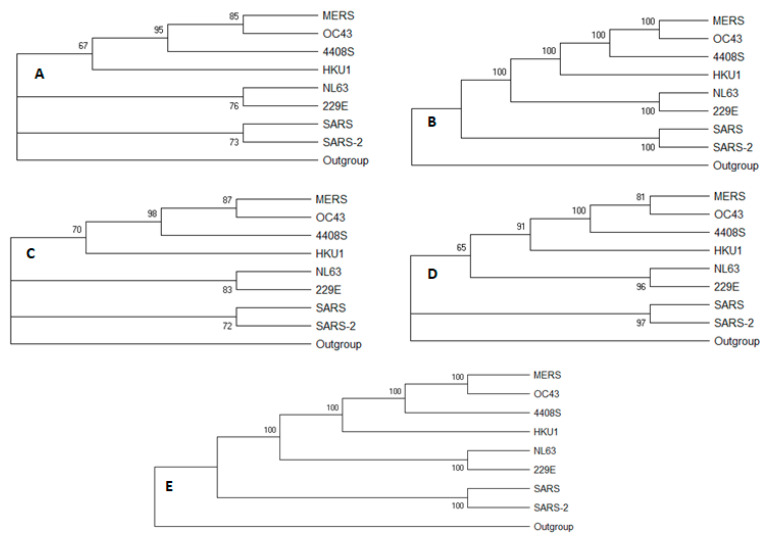
This is diagram shows phylogenetic trees of eight whole genomes of coronavirus species using MEGA7. The evolutionary history for each of the five bootstrap consensus trees (**A**–**E**) was inferred respectively using neighbor-joining, UPGMA, minimum evolution, maximum likelihood, and maximum parsimony methods. The bootstrap consensus tree inferred from 100 replicates and the percentage of replicate trees in which the associated taxa clustered together in the bootstrap test (100 replicates) is shown next to the branches.

In this study, we endeavored to provide a deep understanding of the SARS-CoV-2 through general genomic comparison with seven coronavirus species infecting humans and to a deep level with the closest one. The analysis was performed at the level of genomes, genes, and proteins. Pairwise alignment and evolutionary distance of the eight species have shown that SARS-CoV has the highest identity and the lowest distance in comparison to SARS-CoV-2 (Table 1). Genomic evolutionary trees were constructed using five different methods, with a bootstrapping value of 100 to provide accurate and confident branching, as shown in Figure 1. All methods have shown that SARS-CoV-2 is the most similar to the SARS-CoV species. Our findings support the research findings of Ahmed SF [30], Petrosillo N, and his colleagues [31].

**Figure 2:**
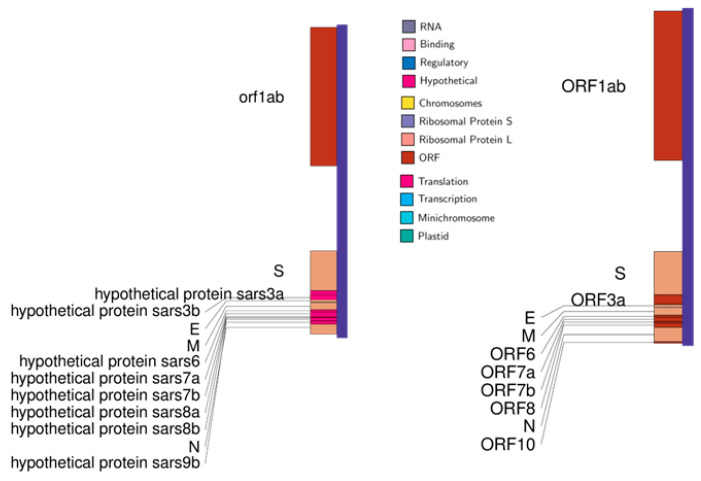
Genomic maps of SARS-CoV on the left and SARS-CoV-2 on the right.

**Figure 3:**
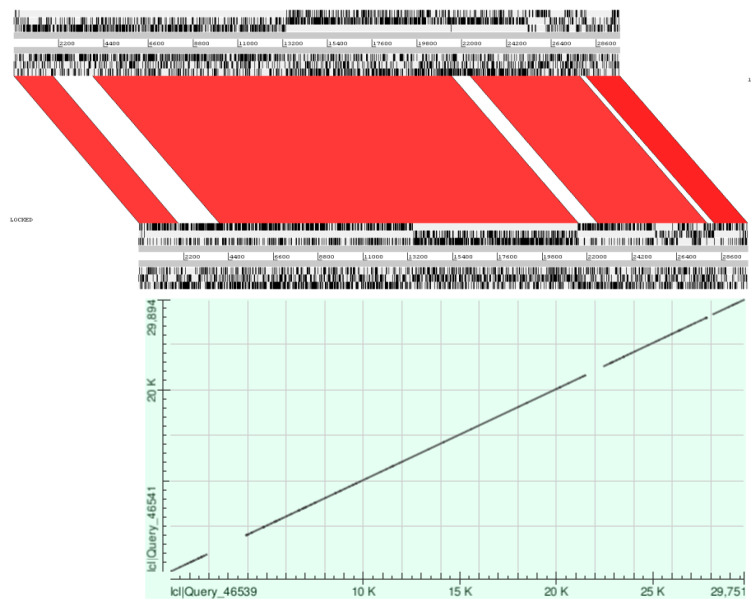
Pairwise alignment of SARS-CoV and SARS-CoV-2 genomes shows similar and nonsimilar regions. Upper illustration shows comparison using Artemis Comparison Tool (ACT). Top and bottom panels view uploaded sequences. In the middle of the sequence view panels is the comparison view. Red blocks link similar regions of DNA, with the intensity of red directly proportional to the level of similarity. Lower illustration shows comparison using BLAST. Dot matrix view shows regions of similarity. The query sequences are represented on the X and Y axes. Alignments are shown in the plot as lines.

Comparative genomic analysis of SARS-CoV-2 with SARS-CoV revealed that the two genomes seemed to have a high similarity; the core genes were shared by both genomes, but there were some genes found in one of them but not in both (three low-match regions) (Figure 2 and Figure 3).

### 3.2. Low Similarity Region Analysis

Some differences existed regarding gene location, sequence, and consequently gene structure, such as the Orf1ab and spike S genes. The genes in three low-match regions were Orf8 in SARS-CoV-2, and Sars8a, and Sars8b in SARS-CoV (Table 2).

**Figure 4:**
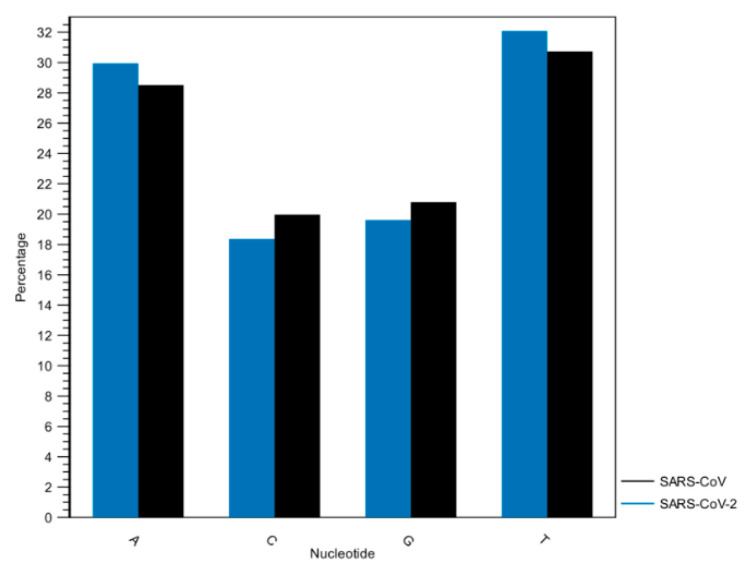
Histogram of nucleotide frequencies of SARS-CoV and SARS-CoV-2 genomes. A = Adenine, C = Cytosine, G = Guanine, T = Thymine.

The variation of genes is partly consistent with the research performed by Shereen MA. et al. [32], who reported the presence of Orf3 protein and absence of Orf10 protein in SARS-CoV-2. Most of the nucleotide sequence statistics presented in Table 3 and Figure 4 (length, molecular weight, number of atoms, and nucleotide frequencies) have also shown that the two genomes are approximately similar. This finding coincides with what Petrosillo et al. mentioned in their study that only minor differences have been found between the genome sequences of SARS-CoV-2 and SARS-CoV [31].

Analysis of region 1 (less similar genomic regions) between the two interested genomes revealed that regions 1 and 2 showed gene identity between 72 and 80 percent. The identity of their protein products ranges from 76 to 86 percent (Table 4).

**Figure 5:**
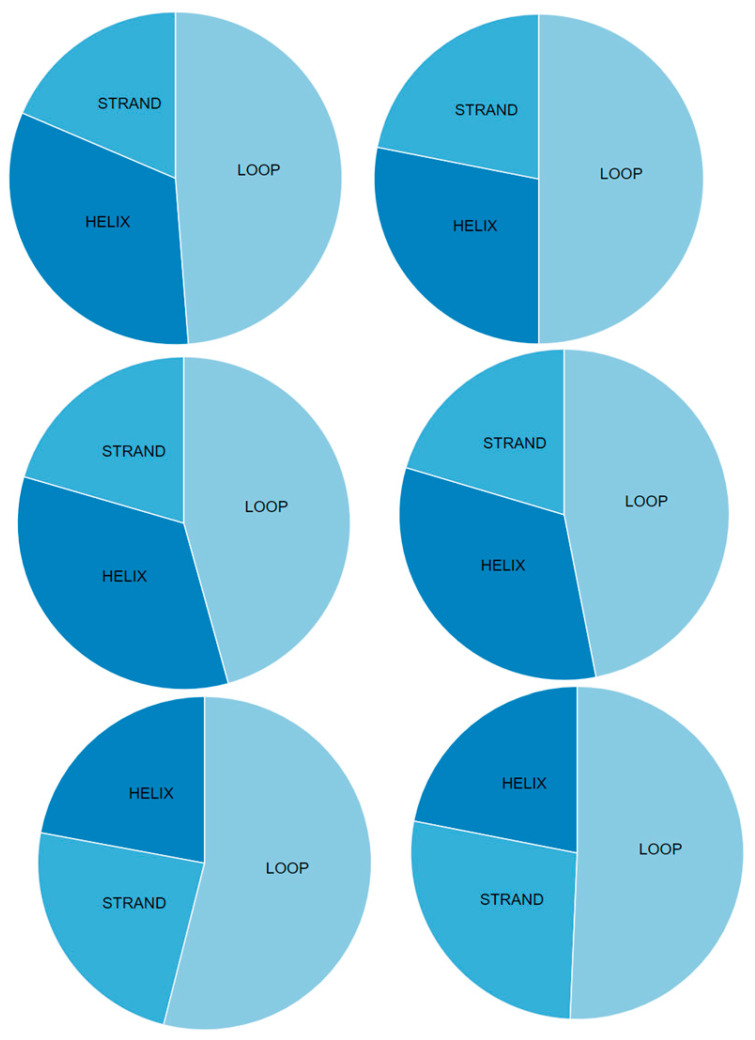
Charts show the secondary protein structures of SARS-CoV (**left**) and SARS-CoV-2 (**right**). Proteins from the top are Orf1ab, Orf1a, and spike, respectively.

**Figure 6:**
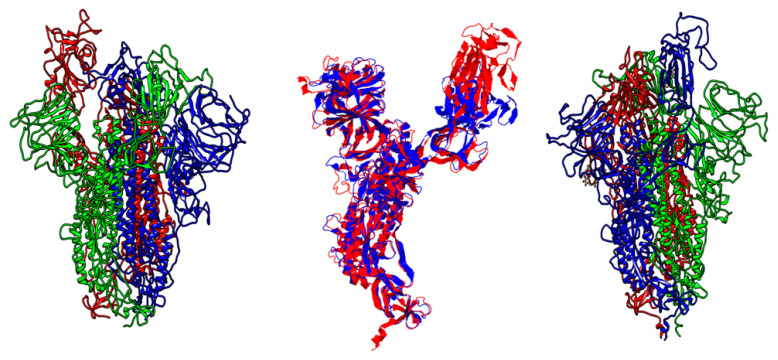
The backbone of spike proteins, in which SARS-CoV is represented on the left and SARS-CoV-2 on the right side. SARS-CoV model was built using template (6acd.1) with 99.92 identities and 0.95 coverage. The structural evaluation scores were as follows: 0.92, 0.80, and −3.25 for QSQE, GMQE, and QMEAN, respectively. By using 6acd.1, the SARS-CoV-2 model was built using a template with 99.26 sequence identities and 0.95 coverage. The structural evaluation scores were as follows: 0.87, 0.72, and −2.81 of QSQE, GMQE, and QMEAN, respectively. Previous structures were constructed using the Swiss-Model server. The central model shows the superposition structures of SARS-CoV in blue and SARS-CoV-2 in red. The aligned length was 971 out of ~1100 residues. Align score was 0.81014 and 4.12 for root-mean-square deviation (RMSD).

**Figure 7:**
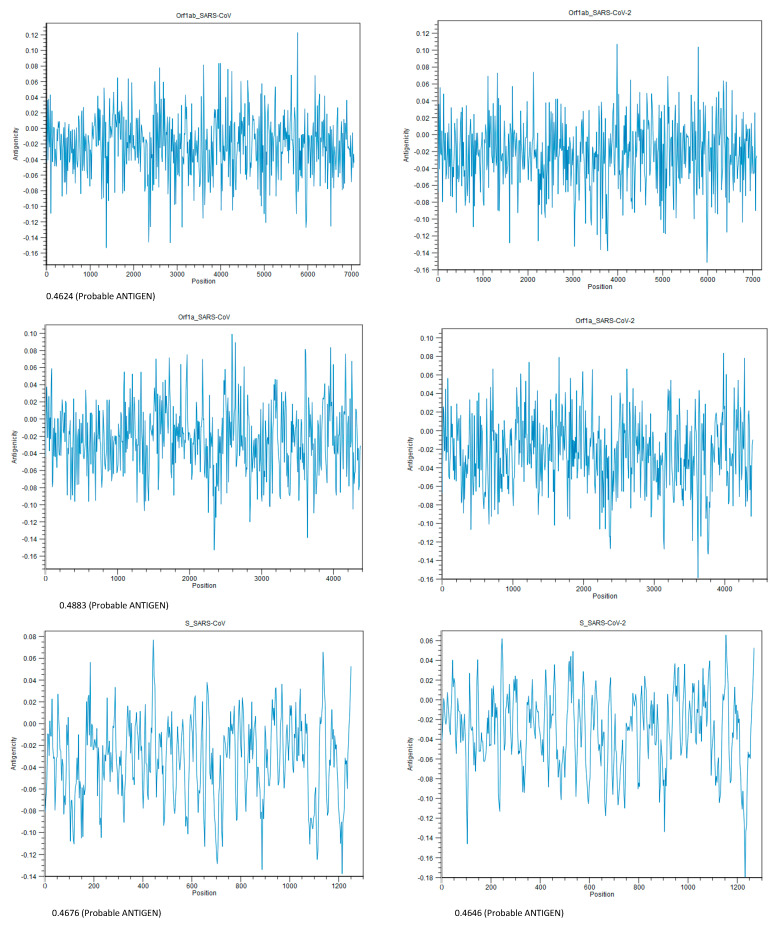
Antigenicity plot of homologous proteins within low-match regions prepared using CLC Genomics Workbench 20.0.3. The number below each plot shows the antigenicity score, using Vaxijen v2.0. The threshold for this model is 0.40. Proteins from the top, Orf1ab, Orf1a, and spike, respectively.

Computational proteomics analysis for nonstructural proteins Orf1ab and Orf1a, and structural S proteins demonstrated the great similarity between the relevant comparative proteins at the primary, secondary, and tertiary structural levels (Table 5 and Table 6, and Figure 5, Figure 6 and Figure 7). These findings reinforce the hypothesis of similarity between these species, and this overlaps with findings achieved by Ceraolo C. and Giorgi FM [33].

From an immunogenic point of view, Orf1ab, Orf1a, and the S proteins of SARS-CoV-2 could be antigenic and capable of exciting the immune system, which means these three proteins could be considered as potential sources of vaccine. The highest score (0.4787) was for Orf1a. The results of the antigenicity test are shown in Figure 7.

The third region contains various genes that are found in one species but not in both, which excludes the possibility of comparison. Genes located in this region are Orf8a(Sars8a) and Orf8b (Sars8b) in SARS-CoV, and Orf8 in SARS-CoV-2 (Table 6). In order to obtain additional information on the protein products of the previous genes, they were compared with a universal database of proteins (UniProt) (Table 7).

**Figure 8:**
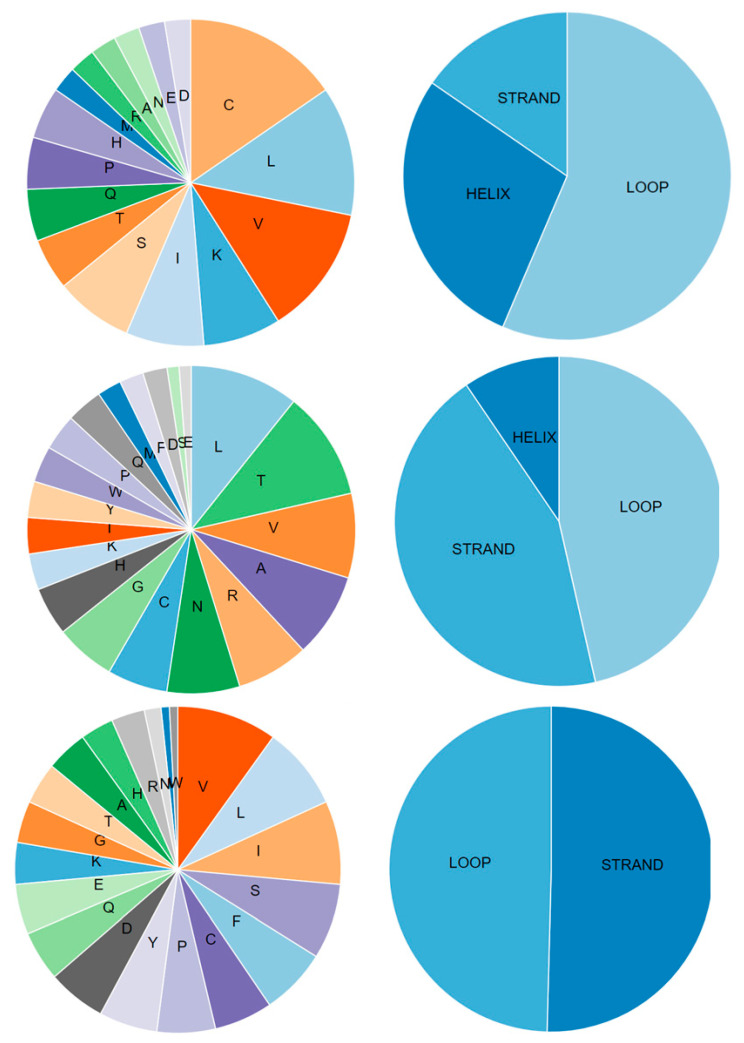
Primary and secondary structures of open reading frames ORF8a, ORF8b, and ORF8 proteins (from **top** to **bottom**). Charts were created using the PROFphd server.

Due to the lack of information in the UniProt database, many additional tools were used. The secondary structure of these proteins was predicted (Figure 8), and the physicochemical parameters were calculated as shown in Table 8.

**Figure 9:**
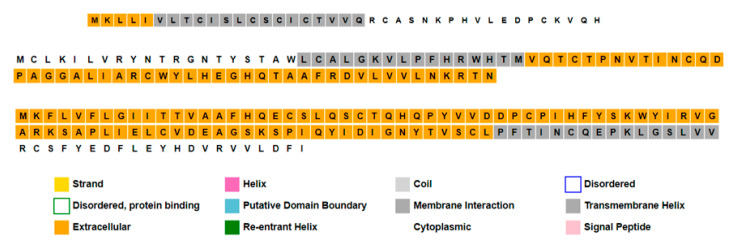
Sequence plots show secondary structure annotation of proteins within the third low-similar region using PSIPRED and MEMSAT tools. Proteins from the top are Orf8a, Orf8b, and Orf8, respectively.

**Figure 10:**
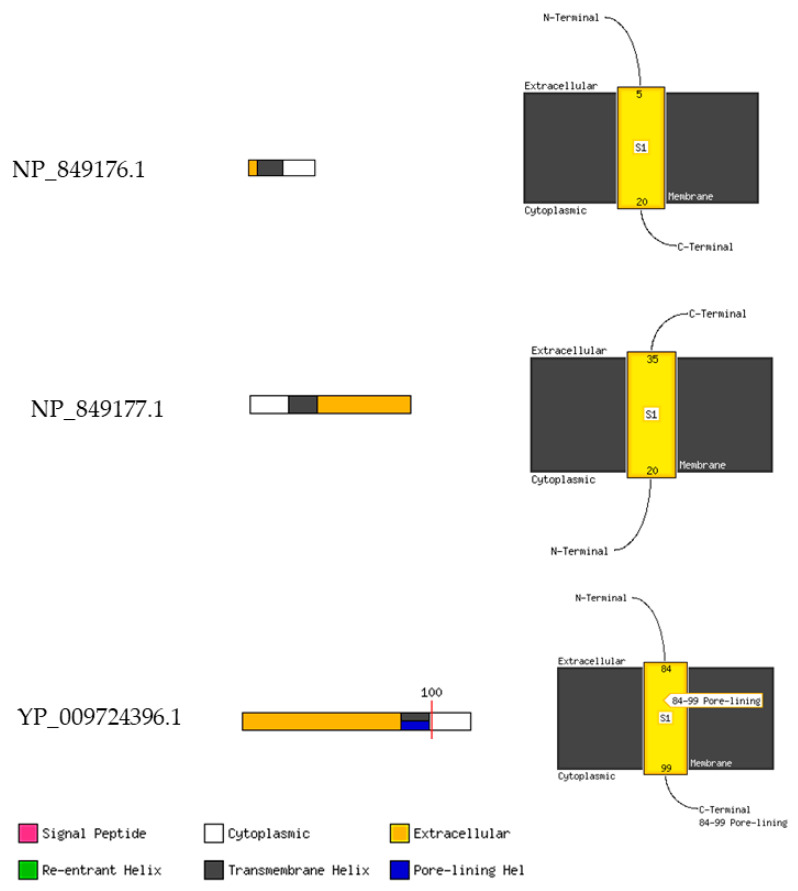
MEMSAT-SVM Schematics. The Diagram on the left shows a cartoon of the MEMSATSVM and MEMSAT3 TM helix predictions. Red line represents the pore-lining helical regions. Further, the right shows the cartoon diagrams of the membrane topology annotated with the predicted helix coordinates.

**Figure 11:**
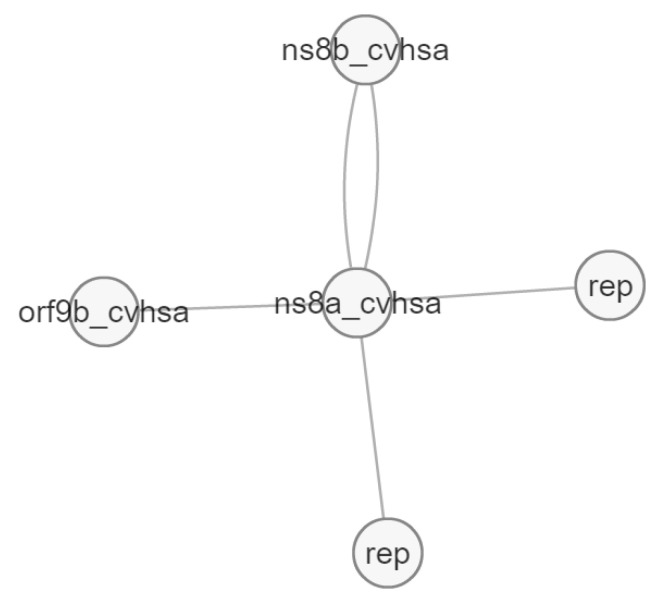
Molecular interactions of Orf8a protein (ns8a).

Annotation of the three proteins predicted that they consist of extracellular, membrane interaction, cytoplasmic, and transmembrane elements (Figure 9, Figure 10 and Figure 11).

Previous findings were consistent with the analysis carried out by Park MD [34]. The predicted functions of these proteins, which are set out in Table 9, were consistent with two studies: the first was conducted by Lau SKP et al., who indicated that Orf8 could be essential for interspecies transmission [35], and the second was accomplished by Keng CT and Tan YJ, who indicated that Orf8a and Orf8b contribute significantly to viral replication and/or in vivo pathogenesis [36,37]. The subcellular locations of these proteins support their predicted roles.

Orf8b and Orf8 could be antigenic and capable of stimulating the immune system (Table 9), and with the highest score (0.6502) for ORF8 among all target proteins in SARS-CoV-2, that means ORF8 protein could be the most promising vaccine against SARS-CoV-2.

**Figure 12:**
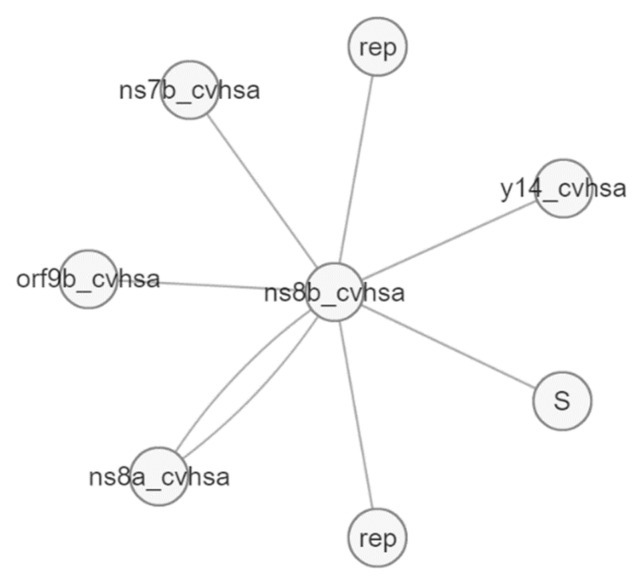
Molecular interactions of Orf8b protein (ns8b).

**Figure 13:**
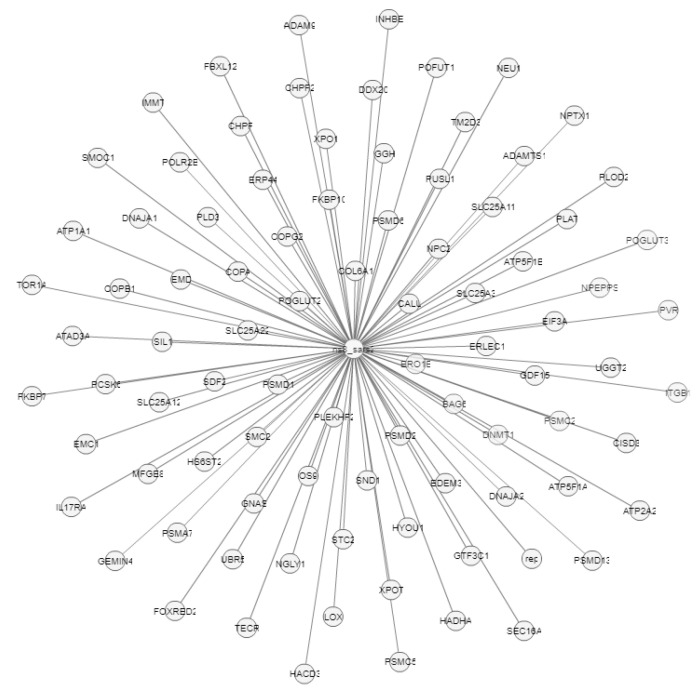
Molecular interactions of Orf8 protein (Ns8).

Figure 12 and Figure 13 presented the interaction of two of the target proteins, and both agreed on the following: (i) interaction between Orf8a/Orf8b, (ii) interaction with proteins that have a role in replication, such as Orf1ab [32], (iii) interaction with proteins that play a role in antiviral signaling and suppressing innate immunity (Orf9b) [38].

The Orf8b protein also has an interaction with the Orf7b protein (ns7b), which contains transmembrane domains that are essential for retaining the protein in the Golgi compartment [39], and the S protein (spike), which facilitates the entry of coronavirus into the host cells [40]. Likewise, Orf8 shows molecular interactions with more than 80 genes, as presented in Figure 13.

**Figure 14:**
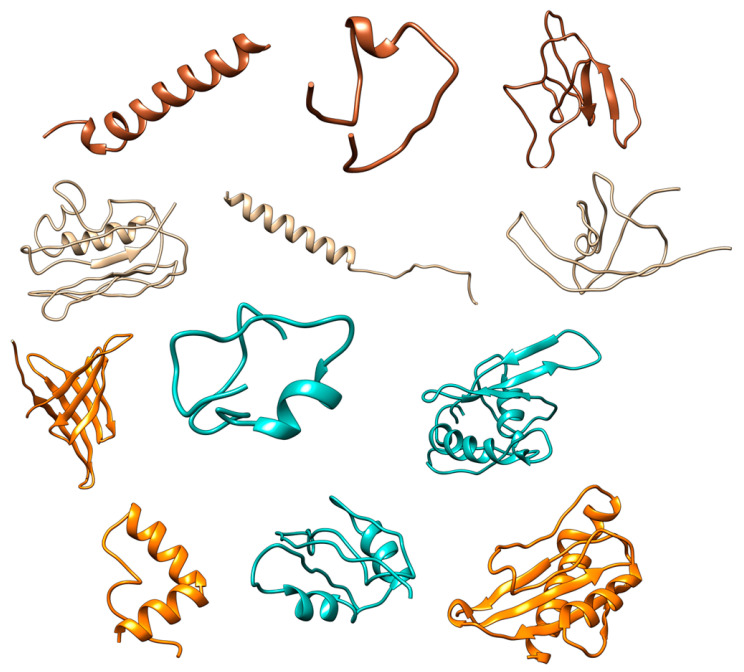
Proteins’ tertiary structure prediction. Proteins from left to right are as follows: Orf8a, Orf8b, and Orf8, respectively. Prediction servers from top to bottom are as follows: Swiss-Model, DMPfold, I-TASSER, and Robetta, respectively.

These molecular interactions are consistent with the proteins’ functions previously expected. Eventually, the protein models predicted by the Robetta server (Figure 14) showed the highest quality score and full-length coverage, as shown in Table 10.

## 4. Conclusions

We concluded that SARS-CoV-2 is the most similar to SARS-CoV among all coronavirus species infecting humans. The core genes were shared by the two genomes, but there were some genes in one of them but not in both, such as ORF8, which is found in SARS-CoV-2 but not in SARS-CoV. The ORF8 protein of SARS-CoV-2 could be considered a good therapeutic target for stopping viral transmission, as it is predicted to be a transmembrane protein, which is responsible for interspecies transmission. ORF1ab, ORF1a, ORF8, and S proteins of SARS-CoV-2 could be immunogenic and capable of exciting the immune system, which means these proteins could be considered potential sources of a vaccine.

The findings of the present study will contribute to the containment of SARS-CoV-2 and may assist other researchers in getting an in-depth understanding and analysis of SARS-CoV-2.

## Figures and Tables

**Table 1:** Pairwise alignment matrix of eight whole genomes of coronavirus species, using CLC Genomics Workbench v20.0.3.

		1	2	3	4	5	6	7	8	9
MERS	1		100.00	97.23	76.28	55.19	55.29	56.99	56.40	55.45
OC43	2	0.00		97.23	76.28	55.19	55.29	56.99	56.40	55.45
4408S	3	0.03	0.03		76.29	55.13	55.23	56.97	56.35	55.50
HKU1	4	0.29	0.2	0.29		54.74	55.98	57.19	57.73	55.67
SARS	5	0.68	0.68	0.68	0.69		81.43	55.85	52.00	51.17
SARS-2	6	0.68	0.68	0.68	0.66	0.21		56.13	52.57	51.22
NEO	7	0.64	0.64	0.64	0.63	0.66	0.65		52.74	52.01
NL63	8	0.65	0.65	0.65	0.62	0.76	0.75	0.74		69.67
229E	9	0.67	0.67	0.67	0.67	0.78	0.78	0.76	0.39	

**Table 2:** Features of low-match regions between SARS-CoV and SARS-CoV-2.

Regions	Features	SARS-CoV	SARS-CoV-2
First	Location	1924–3883	1925–3956
	Genes	Orf1ab	Orf1ab
	Proteins	- Orf1ab - Orf1a	- Orf1ab - Orf1a
Second	Location	21,507–22,429	21,579–22,539
	Genes	Spike S	Spike S
	Proteins	Surface glycoprotein	Surface glycoprotein
Third	Location	27,799–28,103	27,912–28,257
	Genes	- Sars8a - Sars8b	Orf8
	Proteins	- Hypothetical protein Sars8a - Hypothetical protein Sars8b	Orf8 protein

**Table 3:** Nucleotide sequence statistics of the SARS-CoV-2 and SARS-CoV genomes.

Information	SARS-CoV-2	SARS-CoV
Length	29,903 bp	29,751 bp
Weight (single-stranded)	9241.224 kDa	9192.103 kDa
Counts of Atoms		
Hydrogen (H)	368,432	366,157
Carbon (C)	293,538	291,570
Nitrogen (N)	109,749	109,446
Oxygen (O)	180,059	179,169
Phosphorus (P)	29,903	29,751
Comparison elements		
Identities	23,718	
Percent identity	79.12	
Difference	6261	
Gaps	304	
Distance	0.23	

**Table 4:** General and comparative information of genes/proteins found within the first and second low-match regions.

	Descriptions	SARS-CoV ID	Length bp	SARS-CoV-2 ID	Length bp	Identities	Percent Identity	Difference	Gaps	Distance
Name										
Genes										
Orf1ab		1489680	21,221	43740578	21,290	16,972	79.65	4336	105	0.23
Spike		1489668	3768	4374056	3822	2797	72.82	1044	92	0.31
Proteins										
Orf1ab		NP_828849.2	7073	YP_009724389.1	7096	6123	86.20	980	37	0.14
Orf1a		NP_828850.1	4382	YP_009725295.1	4405	3550	80.46	862	37	0.21
Spike (S)		NP_828851.1	1255	YP_009724390.1	1273	974	76.27	303	26	0.25

**Table 5:** Physicochemical parameters of homologous proteins in the first and second low-match regions.

	Proteins	SARS-CoV Orf1ab	SARS-CoV-2 Orf1ab	SARS-CoV Orf1a	SARS-CoV-2 Orf1a	SARS-CoV Spike (S)	SARS-CoV-2 Spike (S)
Descriptions							
Molecular weight		790,248.32	794,057.79	486,372.73	489,988.91	139,109.14	141,178.47
Theoretical pI		6.19	6.32	5.91	6.04	5.56	6.24
Extinction coefficients		920,760 906,260	942,275 928,150	530,660 521,660	552,175 543,550	143,335 140,960	148,960 146,460
Estimated half-life		30 h	30 h	30 h	30 h	30 h	30 h
Instability index		33.65 (stable)	33.31 (stable)	35.51 (stable)	34.92 (stable)	32.42 (stable)	33.01 (stable)
Aliphatic index		87.08	86.87	89.43	88.99	82.80	84.67
Grand average of hydropathicity (GRAVY)		−0.071	−0.070	−0.020	−0.023	−0.043	−0.079

**Table 6:** General information of nonhomologues genes/proteins found within the third low-match regions.

	Descriptions	SARS-CoV ID	SARS-CoV-2 ID	Length	Genome Location	Protein Type (NCBI Database)
Name						
Genes						
Orf8a (Sars8a)		1489676	-	120	27,779–27,898	-
Orf8b (Sars8b)		1489677	-	255	27,864–28,118	-
Orf8		-	43740577	366	27,894–28,259	-
Proteins						
Orf8a		NP_849176.1	-	39	-	Hypothetical protein
Orf8b		NP_849177.1	-	84	-	Hypothetical protein
Orf8		-	YP_009724396.1	121	-	Orf8 protein

All data are retrieved from NCBI database (https://www.ncbi.nlm.nih.gov/, accessed on 2 February 2023).

**Table 7:** Physicochemical parameters of the third low-match regions’ proteins using the ProtParam tool.

	Proteins	Orf8a NP_849176.1	Orf8b NP_849177.1	Orf8 YP_009724396.1
Descriptions				
Molecular weight		4327.30	9560.16	13831.01
Theoretical pI		8.30	9.45	5.42
Total number of negatively charged Total number of positively charged		2 4	3 9	13 9
Extinction coefficients		375 (Low confidence results)	21,220 20,970	16,305 15,930
Estimated half-life				
Instability index		27.07 (stable)	34.68 (stable)	45.79 (unstable)
Aliphatic index		119.74	88.21	97.36
Grand average of hydropathicity (GRAVY)		0.644	−0.029	0.219
Atomic composition				
Carbon (C)		185	425	633
Hydrogen (H)		318	667	961
Nitrogen (N)		52	125	155
Oxygen (O)		52	113	177
Sulfur (S)		7	7	8
Total number of atoms		614	1337	1934

**Table 8:** Comparison of target proteins with the Universal Protein Resource (UniProt).

Subsection	Orf8a		Orf8b		Orf8	
BLASTp results (most significant with 100% of similarity)	Protein nonstructural 8a (UniProt ID: Q7TFA0)		Non-structural protein 8b (UniProt ID: Q80H93)		Non-structural protein 8 (UniProt ID: P0DTC8)	
UniProtKB curators	Reviewed		Reviewed		Reviewed	
Post-translational modifications (PTMs) and/or processing events	Feature key -Signal peptide -Chain	Position(s) 1–15 16–39	Feature key -Chain	Position(s) 1–84	Feature key -Signal peptide -Chain	Position(s) 1–15 16–121
Structure	Nil		Nil		Nil	
Family/Domains or motifs	Corona_NS8/EDPCP and INCQ		Corona_NS8/EDPCP and INCQ		Corona_NS8/EDPCP and INCQ	
Description of proteins’ family	This family of proteins includes the accessory proteins encoded by Orf8 in coronaviruses, also known as accessory protein 8, or nonstructural protein 8 (ns8). Proteins in this ns8 family are typically between 39 and 121 amino acids in length. This protein has two conserved sequence motifs: EDPCP and INCQ. It may modulate viral pathogenicity or replication in favor of human adaptation. ORF8 was suggested as one of the relevant genes in the study of human adaptation to the virus. This entry includes the NS8a and NS8b proteins from the human SARS coronavirus (SARS-CoV).					

**Table 9:** Prediction of proteins’ function, antigenicity, and subcellular location using various resources.

Databases/Server	Orf8a	Orf8b	Orf8
Function			
Pfam database	Nonstructural proteins (8a, 8b, and 8, respectively). This family of proteins is functionally uncharacterized. This protein is found in coronaviruses. Proteins in this family are typically between 39 and 121 amino acids in length. This protein has two conserved sequence motifs: EDPCP and INCQ.		
InterPro database	These proteins have two conserved sequence motifs: EDPCP and INCQ. They may modulate viral pathogenicity or replication in favor of human adaptation. ORF8 was suggested as one of the relevant genes in the study of human adaptation to the virus.		
Conserved Domains database	Fast-evolving proteins in SARS-related CoVs, and a potential pathogenicity factor that evolves rapidly to counter the immune response and facilitate the transmission between hosts.		
PANDA server Biological Process Ontology (BPO) Cellular Component Ontology (CCO) Molecule Function Ontology (MFO)	GPI anchor biosynthetic process (GO:0006506) extracellular region/membrane (GO:0005576/GO:0016020) carbohydrate binding (GO:0030246)	Calcium ion transmembrane transport (GO:0070588) Host cell nucleus/cytoplasm (GO:0042025/GO:0030430) Calcium channel activity (GO:0005262)	Purine ribonucleotide biosynthetic process (GO:0009152) extracellular region (GO:0005576) Calcium ion binding (GO:0005509)
Prosite database	Predicted features: SIGNAL (1- 14) LIPID (15) N-palmitoyl cysteine LIPID (15) S-diacylglycerol cysteine	No feature predicted	No feature predicted
Subcellular Location			
Virus-mPLoc server	-	Host cytoplasm.	Host cell membrane. Host endoplasmic reticulum. Host cytoplasm.
CELLO2GO server (Highest Localization Probability)	Extracellular	Extracellular	Plasmamembrane
Antigenicity (Threshold for this model: 0.4)			
VaxiJen v2.0	0.1251 (Probable NONANTIGEN)	0.5035 (Probable ANTIGEN)	0.6502 (Probable ANTIGEN)

**Table 10:** Evaluation of predicted proteins’ structures using the PROSESS server.

Server Name	Structure	Chain	Helix%	Beta-Strand%	Turn%	Coil%	Protein Length	Overall Quality	Covalent Bond Quality	Non-Covalent/Packing Quality	Torsion Angle Quality
Swiss-Model	Orf8a	L	73%	0%	13%	27%	30	3.5	5.5	3.5	3.5
	Orf8b	A	0%	26%	15%	74%	26	2.5	6.5	3.5	2.5
	Orf8	A	0%	29%	10%	71%	74	3.5	6.5	3.5	2.5
DMPfold	Orf8a	A	56%	0%	10%	44%	39	3.5	5.5	3.5	3.5
	Orf8b	A	5%	41%	4%	54%	84	2.5	3.5	2.5	2.5
	Orf8	A	10%	42%	3%	48%	121	2.5	3.5	2.5	1.5
I-TASSER	Orf8a	A	10%	0%	30%	90%	39	2.5	3.5	3.5	1.5
	Orf8b	A	17%	16%	9%	67%	84	1.5	4.5	3.5	0.5
	Orf8	A	20%	26%	9%	54%	121	2.5	4.5	3.5	1.5
Robetta	3R	A	69%	0%	10%	31%	39	5.5	6.5	6.5	4.5
	7R	A	0%	66%	14%	34%	84	5.5	7.5	6.5	4.5
	11R	A	29%	23%	13%	48%	121	4.5	6.5	5.5	3.5

## Data Availability

The main computational framework is fully described in the paper.

## Abbreviations

CoV: coronavirus; SARS-CoV: severe acute respiratory syndrome; MERS-CoV: Middle East respiratory syndrome; MSA: multiple sequence alignment; MUSCLE: multiple sequence comparison by log-expectation; MEGA7: molecular evolutionary genetics analysis version 7.0; ACT: artemis comparison tool; BLAST: basic local alignment searching tool; QSQE: quaternary structure quality estimate; GMQE: global models quality estimate; QMEAN: qualitative model energy analysis.

## References

[1] Martín-Blanco C., Zamorano M., Lizárraga C., Molina-Moreno V. (2022). The Impact of COVID-19 on the Sustainable Development Goals: Achievements and Expectations. Int. J. Environ. Res. Public Health.

[2] Safitri Y., Ningsih R.D., Agustianingsih D.P., Sukhwani V., Kato A., Shaw R. (2021). COVID-19 Impact on SDGs and the Fiscal Measures: Case of Indonesia. Int. J. Environ. Res. Public Health.

[3] Custodis F., Schwarzkopf K., Weimann R., Spüntrup E., Böhm M., Laufs U. (2020). A SARS-CoV2-negative corona victim. Clin. Res. Cardiol. Off. J. Ger. Card. Soc..

[4] Hassan M.M., Hussain M.A., Kambal S., Elshikh A.A., Gendeel O.R., Ahmed S.A., Altayeb R.A., Muhajir A.M., Mohamed S.B. (2020). NeoCoV Is Closer to MERS-CoV than SARS-CoV. Infect. Dis..

[5] Zhang W. (2020). COVID-19: From Basics to Clinical Practice.

[6] Kahn J.S., McIntosh K. (2005). History and recent advances in coronavirus discovery. Pediatr. Infect. Dis. J..

[7] Abdallah H.M., El-Halawany A.M., Darwish K.M., Algandaby M.M., Mohamed G.A., Ibrahim S.R.M., Koshak A.E., Elhady S.S., Fadil S.A., Alqarni A.A. (2022). Bio-Guided Isolation of SARS-CoV-2 Main Protease Inhibitors from Medicinal Plants: In Vitro Assay and Molecular Dynamics. Plants.

[8] Abdallah H.M., El-Halawany A.M., Sirwi A., El-Araby A.M., Mohamed G.A., Ibrahim S.R.M., Koshak A.E., Asfour H.Z., Awan Z.A., Elfaky M.A. (2021). Repurposing of Some Natural Product Isolates as SARS-CoV-2 Main Protease Inhibitors via In Vitro Cell Free and Cell-Based Antiviral Assessments and Molecular Modeling Approaches. Pharmaceuticals.

[9] Sharma A., Tiwari S., Deb M.K., Marty J.L. (2020). Severe acute respiratory syndrome coronavirus-2 (SARS-CoV-2): A global pandemic and treatment strategies. Int. J. Antimicrob. Agents.

[10] Sifuentes-Rodríguez E., Palacios-Reyes D. (2020). COVID-19: The outbreak caused by a new coronavirus. Bol. Med. Del Hosp. Infant. Mex..

[11] Koley T.K., Dhole M. (2020). The COVID-19 Pandemic: The Deadly Coronavirus Outbreak.

[12] Kavey R.-E.W., Kavey A.B. (2020). Viral Pandemics: From Smallpox to COVID-19.

[13] https://covid19.who.int/.

[14] Zhou H., Chen X., Hu T., Li J., Song H., Liu Y., Wang P., Liu D., Yang J., Holmes E.C. (2020). A Novel Bat Coronavirus Closely Related to SARS-CoV-2 Contains Natural Insertions at the S1/S2 Cleavage Site of the Spike Protein. Curr. Biol..

[15] Malik Y.A. (2020). Properties of Coronavirus and SARS-CoV-2. Malays. J. Pathol..

[16] Yang X.L., Hu B., Wang B., Wang M.N., Zhang Q., Zhang W., Wu L.J., Ge X.Y., Zhang Y.Z., Daszak P. (2015). Isolation and Characterization of a Novel Bat Coronavirus Closely Related to the Direct Progenitor of Severe Acute Respiratory Syndrome Coronavirus. J. Virol..

[17] Chen S., Zheng X., Zhu J., Ding R., Jin Y., Zhang W., Yang H., Zheng Y., Li X., Duan G. (2020). Extended ORF8 Gene Region Is Valuable in the Epidemiological Investigation of Severe Acute Respiratory Syndrome-Similar Coronavirus. J. Infect. Dis..

[18] Wu Z., Yang L., Ren X., Zhang J., Yang F., Zhang S., Jin Q. (2016). ORF8-Related Genetic Evidence for Chinese Horseshoe Bats as the Source of Human Severe Acute Respiratory Syndrome Coronavirus. J. Infect. Dis..

[19] Mousavizadeh L., Ghasemi S. (2021). Genotype and phenotype of COVID-19: Their roles in pathogenesis. J. Microbiol. Immunol. Infect..

[20] Edgar R.C. (2004). MUSCLE: Multiple sequence alignment with high accuracy and high throughput. Nucleic Acids Res..

[21] Kumar S., Stecher G., Tamura K. (2016). MEGA7: Molecular Evolutionary Genetics Analysis Version 7.0 for Bigger Datasets. Mol. Biol. Evol..

[22] Felsenstein J. (1985). Confidence Limits on Phylogenies: An Approach Using the Bootstrap. Evol. Int. J. Org. Evol..

[23] Carver T.J., Rutherford K.M., Berriman M., Rajandream M.A., Barrell B.G., Parkhill J. (2005). ACT: The Artemis Comparison Tool. Bioinformatics.

[24] Yachdav G., Kloppmann E., Kajan L., Hecht M., Goldberg T., Hamp T., Hönigschmid P., Schafferhans A., Roos M., Bernhofer M. (2014). PredictProtein—An open resource for online prediction of protein structural and functional features. Nucleic Acids Res..

[25] Bienert S., Waterhouse A., de Beer T.A., Tauriello G., Studer G., Bordoli L., Schwede T. (2017). The SWISS-MODEL Repository-new features and functionality. Nucleic Acids Res..

[26] Zhang Y., Skolnick J. (2005). TM-align: A protein structure alignment algorithm based on the TM-score. Nucleic Acids Res..

[27] Csaba G., Birzele F., Zimmer R. (2009). Systematic comparison of SCOP and CATH: A new gold standard for protein structure analysis. BMC Struct. Biol..

[28] Buchan D.W.A., Jones D.T. (2019). The PSIPRED Protein Analysis Workbench: 20 years on. Nucleic Acids Res..

[29] Nugent T., Jones D.T. (2009). Transmembrane protein topology prediction using support vector machines. BMC Bioinform..

[30] Ahmed S.F., Quadeer A.A., McKay M.R. (2020). Preliminary Identification of Potential Vaccine Targets for the COVID-19 Coronavirus (SARS-CoV-2) Based on SARS-CoV Immunological Studies. Viruses.

[31] Petrosillo N., Viceconte G., Ergonul O., Ippolito G., Petersen E. (2020). COVID-19, SARS and MERS: Are they closely related?. Clin. Microbiol. Infect..

[32] Shereen M.A., Khan S., Kazmi A., Bashir N., Siddique R. (2020). COVID-19 infection: Origin, transmission, and characteristics of human coronaviruses. J. Adv. Res..

[33] Ceraolo C., Giorgi F.M. (2020). Genomic variance of the 2019-nCoV coronavirus. J. Med. Virol..

[34] Park M.D. (2020). Immune evasion via SARS-CoV-2 ORF8 protein?. Nat. Rev. Immunol..

[35] Lau S.K., Feng Y., Chen H., Luk H.K., Yang W.H., Li K.S., Zhang Y.Z., Huang Y., Song Z.Z., Chow W.N. (2015). Severe Acute Respiratory Syndrome (SARS) Coronavirus ORF8 Protein Is Acquired from SARS-Related Coronavirus from Greater Horseshoe Bats through Recombination. J. Virol..

[36] Keng C.-T., Tan Y.-J. (2020). Molecular and Biochemical Characterization of the SARS-CoV Accessory Proteins ORF8a, ORF8b and ORF8ab. Molecular Biology of the SARS-Coronavirus.

[37] UniProt Consortium (2019). UniProt: A worldwide hub of protein knowledge. Nucleic Acids Res..

[38] Shi C.S., Qi H.Y., Boularan C., Huang N.N., Abu-Asab M., Shelhamer J.H., Kehrl J.H. (2014). SARS-coronavirus open reading frame-9b suppresses innate immunity by targeting mitochondria and the MAVS/TRAF3/TRAF6 signalosome. J. Immunol..

[39] Schaecher S.R., Diamond M.S., Pekosz A. (2008). The transmembrane domain of the severe acute respiratory syndrome coronavirus ORF7b protein is necessary and sufficient for its retention in the Golgi complex. J. Virol..

[40] Li F. (2016). Structure, Function, and Evolution of Coronavirus Spike Proteins. Annu. Rev. Virol..

